# Supplementary figures and images for: Molecular Detection of Leishmania (V.) braziliensis and Leishmania (M.) martiniquensis Infecting Domestic Animals from Panama, Central America
Source: Animals (Basel). 2025 Sep 12;15(18):2677. doi: 10.3390/ani15182677 (PMC12466877; doi:10.3390/ani15182677)

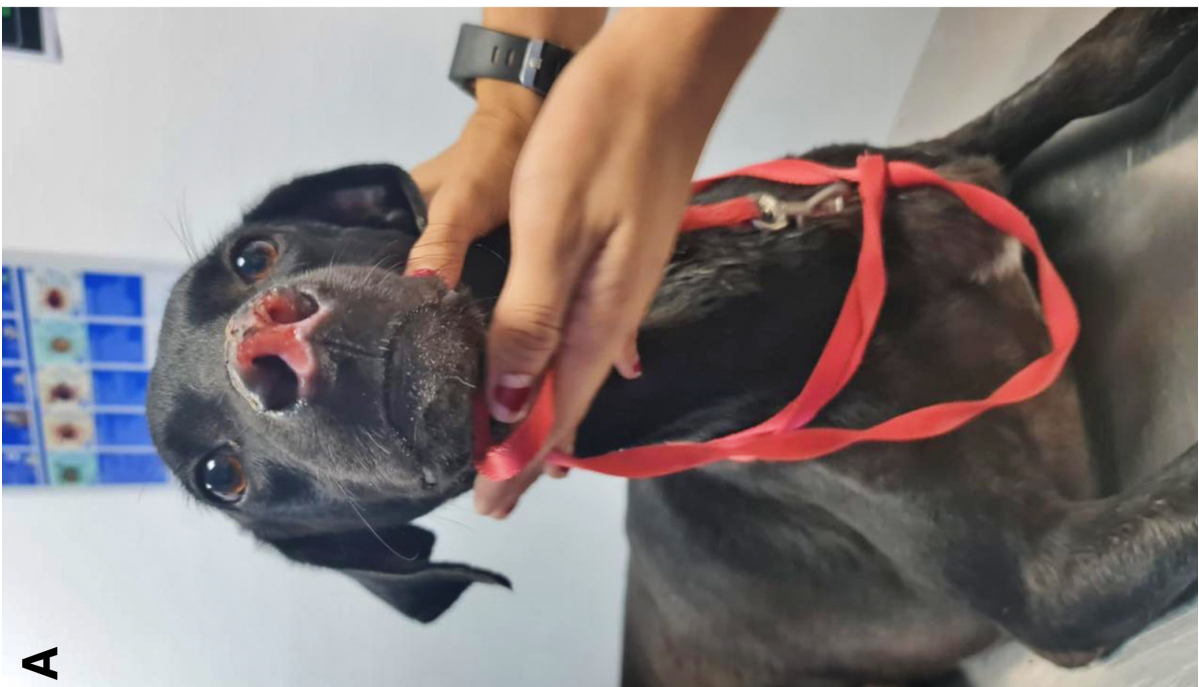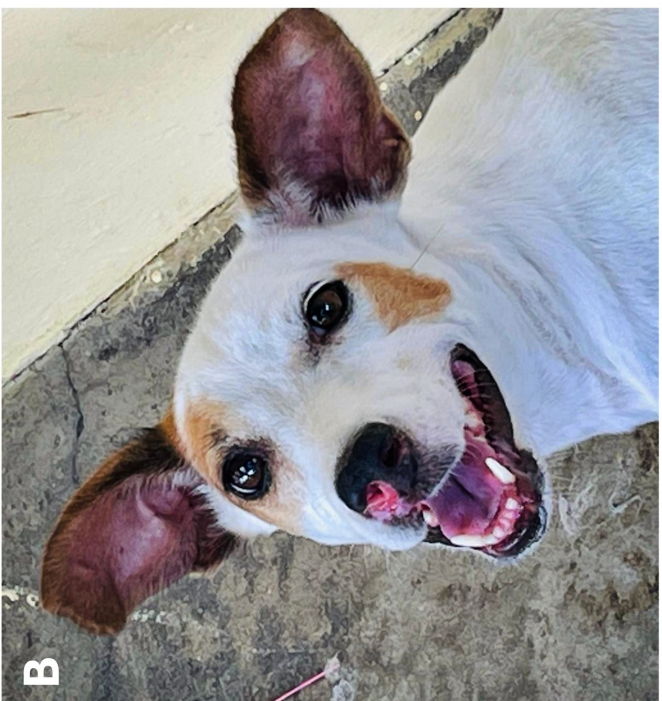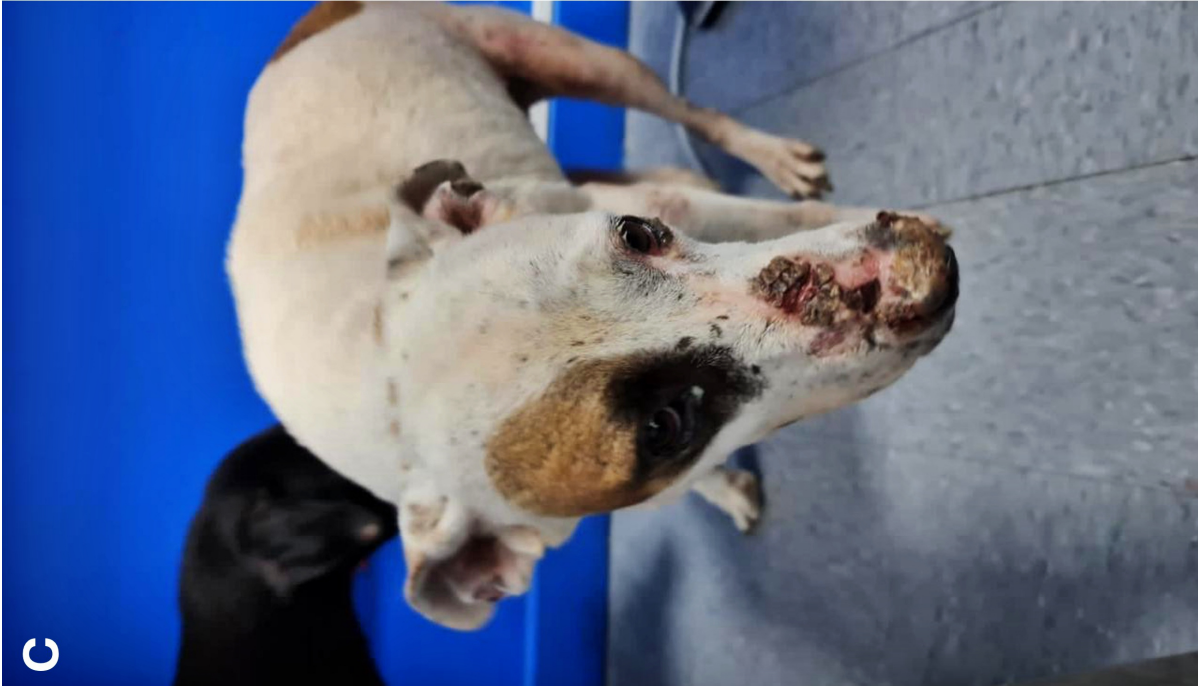

Supplement: Supplementary file 1 [file animals-15-02677-s001.zip › animals-3761331-supplementary.pdf]
